# Simulating dissolved ^90^Sr concentrations within a small catchment in the Chernobyl Exclusion Zone using a parametric hydrochemical model

**DOI:** 10.1038/s41598-020-66623-4

**Published:** 2020-06-17

**Authors:** Yasunori Igarashi, Yuichi Onda, Jim Smith, Sergey Obrizan, Serhii Kirieiev, Volodymyr Demianovych, Gennady Laptev, Dmitri Bugai, Hlib Lisovyi, Alexei Konoplev, Mark Zheleznyak, Yoshifumi Wakiyama, Kenji Nanba

**Affiliations:** 1 Institute of Environmental Radioactivity, Fukushima University, Fukushima, 960-1296 Japan; 2 Center for Research in Isotopes and Environmental Dynamics, University of Tsukuba, Tsukuba, 305-8572 Japan; 3 School of Earth & Environmental Sciences, University of Portsmouth, Portsmouth, PO1 3QL UK; 4 The Chornobyl Radiation and Ecological Biosphere Reserve, Chernobyl, 07270 Ukraine; 5 Chernobyl Ecocentre, State Agency of Ukraine on Exclusion Zone Management, Chernobyl, 07261 Ukraine; 6 Ukrainian Hydrometeorological Institute, Kiev, 03028 Ukraine; 7 Institute of Geological Sciences, Kiev, 01601 Ukraine

**Keywords:** Environmental impact, Hydrology

## Abstract

Strontium-90 (^90^Sr) is the major long-lived radionuclide derived from the Chernobyl accident, and is still being detected in the heavily contaminated catchments of the Chernobyl Exclusion Zone. This study examines the long-term decrease in the dissolved-phase ^90^Sr concentration and the concentration–discharge (^90^Sr*-Q*) relationship in stream water since the accident. We show that the slow decline in ^90^Sr follows a double-exponential function, and that there is a clear relationship between ^90^Sr and *Q*. This study is the first to reveal that the log(^90^Sr)-log(*Q*) slope has been gradually decreasing since the accident. This trend persists after decay correction. Thus, it is not caused by the physical decay of ^90^Sr and environmental diffusion, but implies that the concentration formation processes in stream water have been changing over a long period. We propose a hydrochemical model to explain the time-dependency of the ^90^Sr-*Q* relationship. This paper presents a mathematical implementation of the new concept and describes the model assumptions. Our model accurately represents both the long-term ^90^Sr trend in stream water and the time-dependency of the ^90^Sr-*Q* relationship. Although this paper considers a small catchment in Chernobyl, the conceptual model is shown to be applicable to other accidental releases of radionuclides.

## Introduction

**Figure 1:**
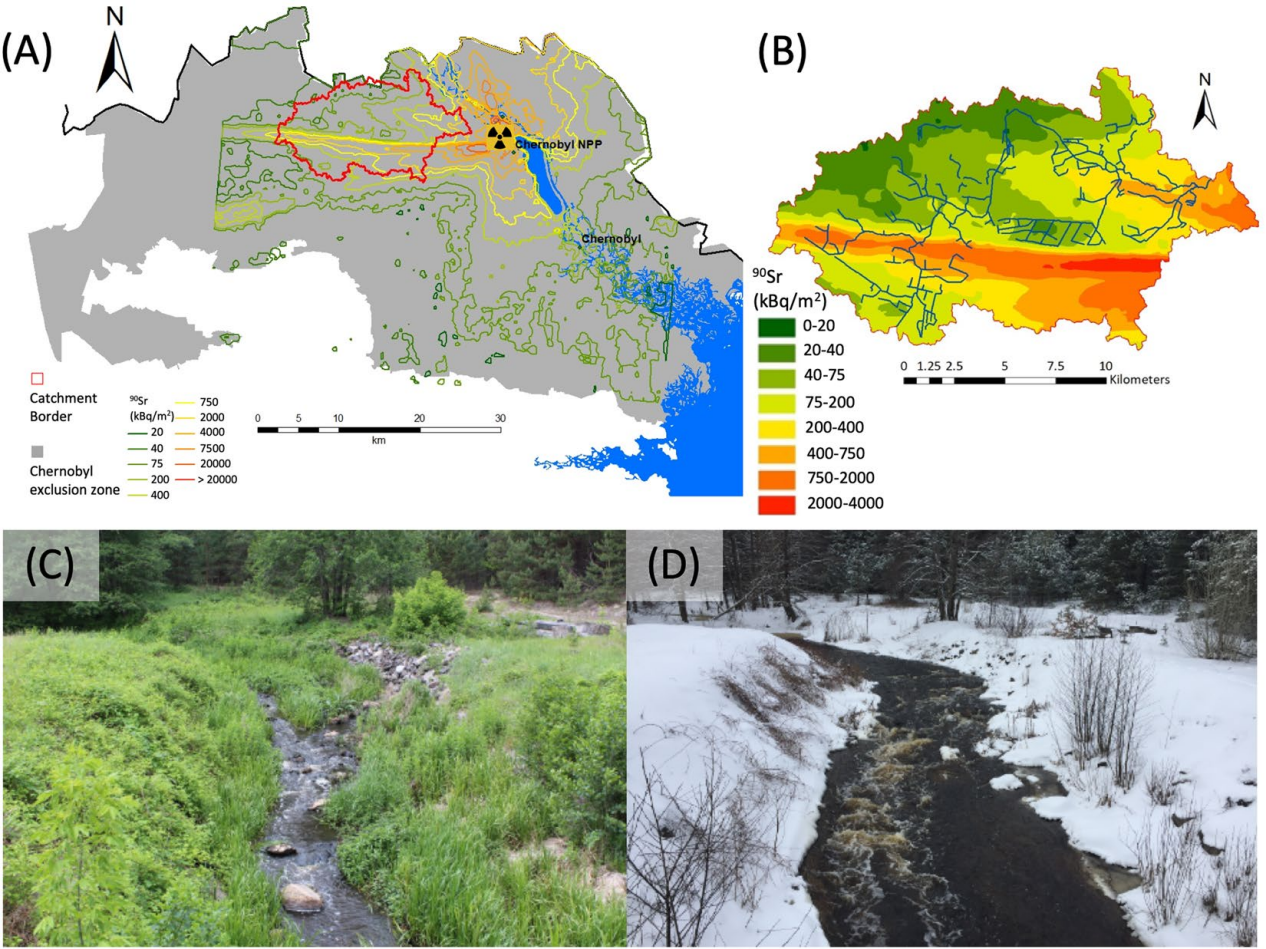
Location of Sakhan river catchment at the Chernobyl Exclusion Zone in northern Ukraine with ^90^Sr inventory in 1997. Flow situation at the Sakhan river mouth in July 2017 (**C**) and February 2018 (**D**).

**Figure 2:**
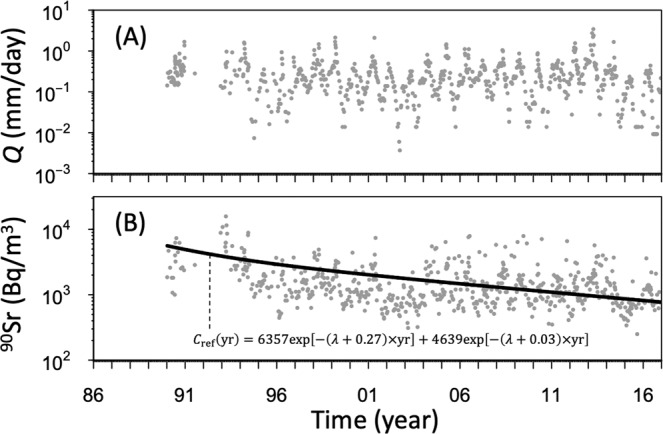
Time series of observed discharge (*Q*: mm/day) (**A**), dissolved-phase ^90^Sr concentration (Bq/m^3^) (**B**).

It has been 34 years since the Chernobyl nuclear power plant (CNPP) accident in Ukraine (Fig. 1), when large amounts of radionuclides were released into the environment. At present, high levels of dissolved-phase strontium-90 (^90^Sr) are still being detected in the small catchment streams inside the Chernobyl Exclusion Zone (CEZ) (Fig. 2b). The main long-term source of exchangeable and available ^90^Sr in the CEZ soils is micron-sized “fuel particles” (FP) that are gradually dissolving. These are derived from the accidental release from CNPP Unit 4 on 26 April, 1986, and were formed by the mechanical destruction of nuclear fuel. The dissolution rate of FP in CEZ soils varies from several percent to several tens of percent of the activity inventory per year, depending on the degree of oxidation of the particle UO_2_ matrix and the ambient geochemical conditions (e.g. soil solution pH and oxygen availability)^1–6^. The ^90^Sr from small streams in the CEZ is discharged into the main Pripyat river, which is a critical component of the Dnieper river–reservoir system, one of the largest surface water systems in Europe^7^. Through this river system, ^90^Sr is also transported to the Kiev metropolitan area in Ukraine. Therefore, predicting and evaluating the radionuclide flux within the water system, and using models to evaluate the potential radiological consequences to the downstream population, remain important tasks.

The annual-scale dissolved ^90^Sr concentration in the CEZ rivers is known to exhibit a rapid decrease in recently deposited activity followed by slower transfers during subsequent years, and this can be adequately expressed by an exponential function^8–10^. At the same time, the ^90^Sr concentration varies widely over the course of a year. For instance, the ^90^Sr concentration increases with increasing water levels in the Pripyat river during the snowmelt season^11,12^. The short-term temporal variations in ^90^Sr concentration in the CEZ rivers have been expressed using two- and three-dimensional numerical simulations^13–15^. The differential equations governing the water and sediment transport, and the calculation of radionuclide concentrations using the distribution coefficient (*K*_d_) between sediment and water, have been used in short-term simulations.

The relationship between dissolved ions and/or other substances and the discharge rate (*C*-*Q* relationship) is a general phenomenon that has been widely observed for soluble ions and dissolved organic carbon in various river water systems worldwide^16–23^. The tight coupling between ^90^Sr and the stream discharge rate (*Q*) in CEZ rivers can be explained by the empirical and theoretical background and parametric modelling developed in previous hydrochemical studies^24–26^. Based on these studies, the variations in stream chemistry with respect to discharge can be explained by the variations in water and solute fluxes with respect to depth for a representative soil profile^19,22^. Here, the source of ^90^Sr observed in the CEZ rivers is the oxidized/non-oxidized FP released by the accident^2–5^. The ^90^Sr dissolved from the FP is thought to migrate into the soil before being transported by the water flux, finally reaching the river water. The difference between ^90^Sr in Chernobyl and the dissolved ions observed in other catchments is that the soil solute concentration profile (in this case, vertical ^90^Sr concentration profile in soil) changes over time. Thus, the ^90^Sr-*Q* relationship is assumed to change with time. Therefore, the goal of this study is to examine the long-term ^90^Sr concentration formation process by focusing on the ^90^Sr-*Q* relationship and to develop a conceptual model and mathematical implementation that explain the observed data in the field. The analysis was performed using long-term field measurement data collected biweekly from the Sakhan catchment in the CEZ (Fig. 1). The results of this study will not only inform better conceptual models for accident-derived radionuclide concentrations in rivers, but also provide new concepts regarding the dissolved ion concentration formation process in rivers.

## Theory

Based on the clear relationship between ^90^Sr and *Q* from Chernobyl^24–26^, we assumed that the ^90^Sr-*Q* relationship in Chernobyl rivers could be described by the parametric *C*-*Q* model^16–23^. As the *C*-*Q* relationship, which is primarily determined by the solutes produced from mineral weathering/leaching, is governed by geochemical processes and climate, it is assumed to be in a steady state. It is also assumed that the radionuclide concentrations in the local streams are affected by weathering/leaching processes^1–5^ in the environment. For instance, the radioactive half-life of ^90^Sr is 28.79 years, which is too fast to assume a steady state in the *C*-*Q* relationship. Thus, the time series of observed dissolved ^90^Sr concentrations should be corrected by the long-term diminishing trend. The long-term diminishing trend of ^90^Sr concentrations (*C*_ref_: Bq/m^3^) in the stream is expressed as follows:

1 $${C}_{{\rm{ref}}}(t)=\alpha \exp [-({\lambda }+{k}_{1})\times t/365.25]+\beta \exp [-({\lambda }+{k}_{2})\times t/365.25]$$

where *t* (days) is the number of days after the Chernobyl accident (26/4/1986), *λ* (1/yr) is the physical decay rate (= 0.024) of ^90^Sr, and *α*, *β* (Bq/m^3^), *k*_1_, and *k*_2_ (1/yr) are empirically determined (radionuclide-specific) constants. An exponential model from previous studies^8–10^ was used to describe the decrease in ^90^Sr concentrations over decadal time-scales. The constants *α*, *β*, *k*_1_, and *k*_2_ were set so as to minimize the root mean square error (RMSE) between the annual averaged values and the predicted values using the ‘optim’ package in the R software^27^. The annual averaged values were used to avoid excessive fluctuations from non-averaged data in the model validation stage.

### Igarashi model

This is a new conceptual model that derives a *C*-*Q* relationship by developing the idea of a vertical distribution of the lateral flow of water across the vertical profile of soil water chemistry near the river^19,22^. The key assumption in our model is that the distribution of the lateral flow of soil^22^ and the vertical profile of radionuclides in the soil provides a stream discharge and determines the radionuclide concentrations in a stream. The radionuclide concentration profile in the soil is then represented by the following modified equation:

2 $$c(z,t)={C}_{{\rm{r}}{\rm{e}}{\rm{f}}}\exp \left(\frac{dz}{t}\right)$$

where *c*(*z*, *t*) is the concentration as a function of depth *z* and time *t* since fallout on the surface (26/4/1986). *C*_ref_ is the long-term diminishing trend of ^90^Sr concentration, and *d* is a model coefficient. The *z*-axis has negative values below the ground surface, and intersects the ground surface at *z*_0_. Thus, the ^90^Sr concentration in the stream is defined by an integral expression, which can be solved analytically to give:

3 $$C(Q,t)=\frac{{C}_{{\rm{r}}{\rm{e}}{\rm{f}}}bt}{bt+d}{\left(\frac{bQ}{a}\right)}^{d/bt}$$

This type of power-law function also represents an empirical method of describing the log(*C*)-log(*Q*) relationship. It has been clarified mathematically that the relationship between ^90^Sr and *Q* in a stream obeys a log-log relationship. In this study, the model parameters were calibrated against observed ^90^Sr concentration data. The objective function implemented in the Markov chain Monte Carlo modelling is the standard log-likelihood function. Details can be found in the Supplementary Information.

## Results

### Long-term decline and seasonal fluctuation of ^90^Sr concentration in CEZ streams

The long-term trends of ^90^Sr and *Q* are shown in Fig. 2(a,b). Unfortunately, data for *Q* and the ^90^Sr concentration were not available for the four years following the accident. Thus, we focused on the long-term time series in ^90^Sr and *Q*. Firstly, we found that the long-term data indicate large seasonal variations in *Q* (Fig. 2a). High values of *Q* were observed from March–May each year, which is defined as the snowmelt season, with the highest *Q* recorded on April 13, 2013 (*Q* = 4.2 mm/day). After the snowmelt season, *Q* began to decrease and stayed relatively low during the summer. The lowest *Q* was recorded between August 4 and December 5, 2015 (*Q* = 0.004 mm/day). ^90^Sr concentrations exhibit a long-term decline following a double-exponential function. We applied the exponential function to the ^90^Sr concentration data measured from 1990–2016 (Fig. 2b). The fitted parameters of the exponential model are *α* = 9357 (Bq/m^3^), *β =* 2500 (Bq/m^3^), *k*_1_ = 0.254 (1/yr), and *k*_2_ = 0.002 (1/yr). A large seasonality was also observed in the long-term decrease in ^90^Sr concentrations. High ^90^Sr values were observed during the snowmelt season, and ^90^Sr decreased during the summer. Here, the mean observed ^90^Sr concentrations in stream water decreased from ~3340 (Bq/m^3^) in the period 1990–1995 to ~1450 (Bq/m^3^) from 2012–2016. Using the observed ^90^Sr concentrations and *Q*, the wash-off of ^90^Sr through the stream discharge (from 1993–2016) was estimated to be ~5.0 (kBq/m^2^). This is only 1% of the initial ^90^Sr inventory across the whole watershed.

### Response of ^90^Sr concentration to stream discharge rate

**Figure 3:**
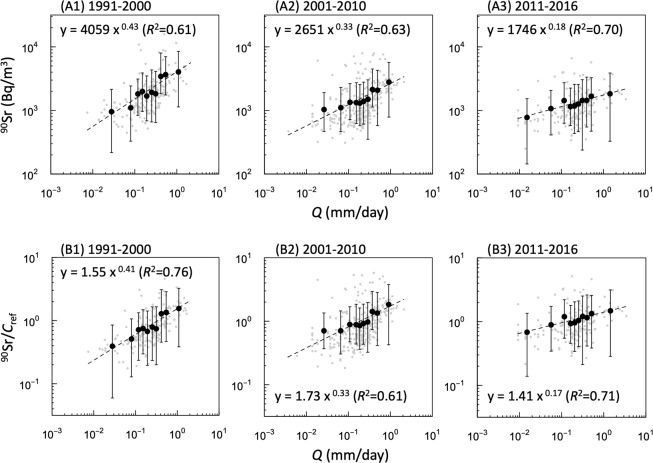
Response of dissolved-phase ^90^Sr concentrations (**A**) and ^90^Sr/*C*_ref_ (**B**) to discharges rate. (1), (2), and (3) for each panel indicate the period used for aggregation, 1991–2000, 2001–2010, and 2011–2016, respectively. Grey dots indicate the observed values. Black dots and bars represent averages and quantiles for observed ^90^Sr, divided into 10 classes by the x-axis. Dashed lines express the best-fitted power-law regression lines for each period.

The relationship between ^90^Sr concentration and *Q* is shown in Fig. 3. A power-law function (*y* = *px* ^*q*^) was fitted for each period (Fig. 3), and the parameter *p* was found to decrease according to the physical decay of ^90^Sr and diffusion in the environment; the power-law exponent *q* (slope of log(^90^Sr)-log(*Q*)) also decreased over time (Fig. 3, a1–a3). This study is the first to show the time-dependency of the ^90^Sr-*Q* relationship from long-term field observations in the Sakhan catchment. When the ^90^Sr concentration was corrected according to its long-term attenuation (^90^Sr/*C*_ref_), the constant parameter *p* was found to range from 1.41–1.73, but the power-law exponent *q* continued to decrease even after the attenuation correction (Fig. 3, b1–b3). This result means that the time-dependency of the ^90^Sr-*Q* relationship is not caused by the long-term attenuation of the ^90^Sr concentration, but by the changing concentration formation processes over a long period of time.

## Discussion

### Long-term decline of ^90^Sr concentration

The contamination of stream water by ^90^Sr continues to pose problems in the CEZ. Long-term monitoring and simple modelling are needed to ensure the safety of water usage in downstream areas and to further understand the dissolved ^90^Sr dynamics. For this study, we obtained data from continuous observations of dissolved-phase ^90^Sr concentrations and stream water discharges from 1990–2016 in the Sakhan catchment, located northwest of CNPP. We then applied a double-exponential model to the long-term ^90^Sr data. As shown in Fig. 2b, we were able to model the long-term decline in the ^90^Sr concentration. The best-fit parameter values for *k*_1_ and *k*_2_ were found to be 0.27 (1/yr) and 0.03 (1/yr), respectively. Note that our ^90^Sr data start from 1990, five years after the accident. In general, exponential functions for the decay of radionuclide concentrations in rivers are thought to consist of a fast “flush” of activity as a result of rapid wash-off processes, a slow decline as a result of soil fixation and redistribution processes, and a very-long-term “equilibrium” situation^8,9^. According to Sasina *et al*.^10^, the fast flush phase of ^90^Sr concentrations originating from the Chernobyl accident in Belarusian rivers lasted for about 0.04 years after the accident (there is a distance of about 90 km from CNPP to the nearest point). Thus, the fast flush of activity as a result of rapid wash-off processes would already have finished by the earliest monitoring year of 1990, and so the values obtained for *k*_1_ and *k*_2_ represent the slow decline as a result of soil fixation and redistribution processes and the very-long-term equilibrium situation. For ^90^Sr deposited in rivers by global fallout (e.g. from atmospheric nuclear weapons testing), the slow decay after the initial sharp drop in concentration is represented by a parameter value of 0.09 (1/yr)^9^. The long-term attenuation component calculated from a study of ^90^Sr in five Belarusian rivers was also estimated to be 0.09 (1/yr)^10^. From our results, the long-term reduction in ^90^Sr concentrations in the Sakhan catchment (*k*_1_ = 0.27 (1/yr)) is three times higher than the rates reported for other rivers only 90 km away. Other rivers in Belarus are also located in the Belarusian Chernobyl area, and it is unlikely that *k* has been affected by climatological differences. This finding may be attributed to the fact that the main source of ^90^Sr in the Sakhan catchment soils is non-oxidized FP that is dissolving relatively slowly;^2–5^ this forms the “Western trace” of Chernobyl contamination across the Sakhan watershed. The dissolution rate of ^90^Sr from non-oxidized FP is 7–10 times slower than from oxidized FP, and model estimates^2^ show that, in 2016, the main portion of radionuclides (up to 47% at pH = 6.0) was still within the particle matrix in the Western trace, whereas the basic portion of FP had dissolved by this time at other points. Therefore, the slow dissolution of FP (which has already disappeared from other basins) incorporating ^90^Sr in the Sakhan catchment could lead to the higher *k* value compared with the other regions mentioned above. Overall, *k* is an integral parameter that can be influenced by both hydrological run-off processes and geochemical factors (controlling the leaching of ^90^Sr from FP and redistribution in soils) in the catchment. Further studies are needed to clarify this issue.

### ^90^Sr-*Q* relationship and its time-dependency

An accurate description of the response of the ^90^Sr concentration to *Q* is another important mission in this study. The variation of ^90^Sr corresponded to increases in *Q* during the snowmelt season and subsequent decreases in summer (Fig. 2b). The recurring annual pattern in *Q*, which rises in the spring and falls in the summer, is clearly influenced by snow melting in spring^11,25,26^ and the increase in evapotranspiration in summer^28^. As for the ^90^Sr-*Q* relationship, studies have shown that ^90^Sr increases when the flow rate of the main stream of the Pripyat River increases during the snowmelt period^14,15^, and ^90^Sr concentration have also been found to increase with spring floods in small catchments under the influence of discharge levels^25,26^. Interestingly, the normalized surface wash-off coefficient for dissolved-phase ^90^Sr (*N*_l_(^90^Sr)) during summer rainfall events is almost twice that of the snowmelt season^1^. This is because ^90^Sr exchange between wash-off liquid and topsoil cannot occur when the topsoil layer is frozen^29^. However, as reported in previous studies^14,25,26^, high ^90^Sr concentrations appear in high-*Q* periods (i.e. snowmelt season, heavy rainfall). Here, the discharge process could be the key to understanding the formation of solutes in the river. As shown by Freed *et al*.^25,26^, there is a clear correspondence between the stream flow and the groundwater level, and in the Chernobyl area, which is characterized by small differences in elevation within the basin, small increases in the groundwater level would cause large increases in the area of saturated soils. A relatively large contribution from the near-surface layer could then recharge the discharge level, leading to high dissolved-phase ^90^Sr concentrations in the snowmelt season. In addition, the effect of *N*_l_(^90^Sr) on the ^90^Sr concentration in the river could be limited, because this region is too flat for the wash-off liquid to reach the river^30^. Frequent sampling could reveal a link between heavy rainfall events and ^90^Sr wash-off during the summer, ﻿therefore, requires further study.

Another key finding of this study besides the clear ^90^Sr-*Q* relationship is the time-dependency of the ^90^Sr-*Q* relationship from long-term field observations (Fig. 3a). It is also worth noting that the time-dependency of the ^90^Sr-*Q* relationship is preserved after the attenuation correction of ^90^Sr (Fig. 3b). Clearly, the log(^90^Sr/*C*_ref_)-log(*Q*) slope during the first 10-year period (1991–2000) is greater than that in the last six years (2011–2016). If the ^90^Sr concentration in the stream is determined by the interaction between water and solutes in the soil, the time-dependency of the ^90^Sr-*Q* relationship is presumably the result of a temporal change in the solute concentration in the soil^19,22^. This is because the water flow pathways in the soil do not change over timescales of a few decades. The solute concentration in the soil, which is the source of ^90^Sr in the stream water, is thought to have changed over time just after the accident. In fact, the vertical profile of the ^90^Sr concentration in the soil changes with time. This is the result of leaching from the initially deposited FP and subsequent downward movement in the soil^1,31^. Various previous studies have discussed the *C-Q* relationship. In particular, previous attempts to introduce parametric models and explain the *C-Q* relationship conceptually could help us to understand the time-dependency of the ^90^Sr-*Q* relationship.

### New parametric model of ^90^Sr concentration

**Figure 4:**
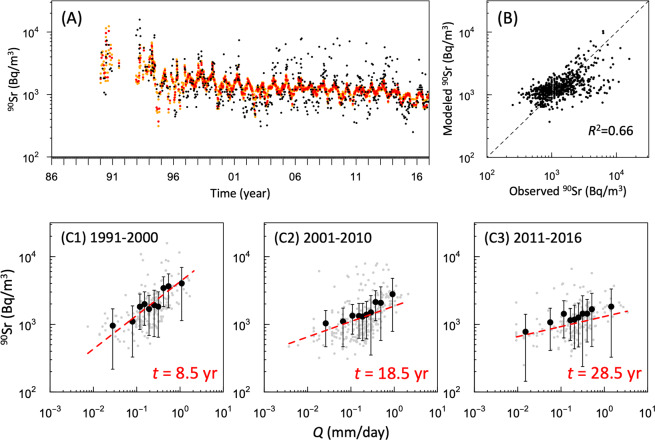
Time series of observed (black) and modelled values (red) of dissolved-phase ^90^Sr concentrations. Orange dots indicate the posterior predictive credible interval (**A**). The relationship between observed and modelled values of dissolved-phase ^90^Sr concentrations. The response of dissolved phase ^90^Sr concentrations to discharges rate for each period are also shown in (C1-3). Red dashed lines in (**C**) indicate the model result for the middle of each period.

A previous hydrochemical study^19^ showed that the stream water chemistry can be accurately explained by a conceptual model that builds on the interaction of the vertical profiles of lateral water fluxes and soil solution chemistry. The mathematical implementation of this conceptual model provides an analytical solution, and a physical explanation as a power function, which is empirically used to describe the relationship between concentration and discharge^22^. Here, we propose a new conceptual hydrochemical model to explain the time-dependency of the ^90^Sr-*Q* relationship. The results of our model (Eq. (4)) are compared against measurements for 1990–2016 in Fig. 4a. Our model shows that the long-term diminishing trend of ^90^Sr is caused by the physical decay of ^90^Sr and diffusion in the environment, as well as the variation of ^90^Sr concentration corresponding to changes in *Q*. Table 1 reports the best fitting parameter with the range of credible values and the agreement between the modelled and observed ^90^Sr concentrations. Regardless of some uncertainty introduced by measurement errors, the model is sufficiently reliable to link ^90^Sr and *Q* for the purposes of this work (*R*^2^ = 0.66). Figure 4c shows the response of ^90^Sr to *Q* for three periods (C1: 1991–2000, C2: 2001–2010, C3: 2011–2016), and the red dashed lines show the model result for the middle of each period. Our model also adequately reproduces the time-dependency of the ^90^Sr-*Q* relationship for each of these periods (Fig. 4c). The key assumption in our model is that the distribution of the lateral flow of soil and the vertical profile of radionuclides in the soil provides a stream discharge and the radionuclide concentration in a stream. ﻿This assumption is supported by the following physical processes observed in the CEZ. During high-flow events (i.e. snowmelt, heavy rainfall), ^90^Sr is directly leached from the contaminated near-surface soil and/or top layer of floodplain soils in the CEZ^14^. The resulting high groundwater table produces lateral flow and increases the chances that more water will pass through the highly contaminated surface soil layer. This highly contaminated soil water enters streams and rivers, leading to an increase in both flow rate and ^90^Sr concentration in watercourses. Studies conducted in the Borschi watershed of the CEZ^25,26^ indicate that near-channel wetland areas can act as a source of ^90^Sr to surface water. In this study, we assumed that the ^90^Sr concentration profile in the soil is represented by an exponential function with a time component. Actually, the vertical profile of the ^90^Sr concentration in soil changes with time according to vertical migration^1,31^ and transport by the water flow^25,26^. It has been reported that ^90^Sr had accumulated in deeper areas (more than 10 cm deep) and was depleted nearer the surface (~5 cm depth) of wetland sediments near the stream some 14 years after the accident^26^. However, we also need to focus on the source of ^90^Sr in the catchment. As shown by Ivanov *et al*.^32^, the peak ^90^Sr concentration was on the soil surface in the Sakhan catchment, even 21 years after the accident. This is because the source of ^90^Sr in Sakhan is non-oxidized FP with a slow dissolution rate^2–5^. Thus, these previous studies support the use of Eq. (3) to represent the temporal changes in the vertical profile of the ^90^Sr concentration in soil. In addition, our model structure, in which the concentration converges to zero at low flow rates (*Q* → 0), is justified by the actual hydrological processes. This is because the source of the river water is assumed to be deep groundwater when *Q* is asymptotically close to zero, and the ^90^Sr concentration, which originates from FP, is assumed to be low, so the ^90^Sr concentration eluted from the groundwater can be assumed to be close to zero.

**Table 1:** Estimated model parameters and model agreement.

Model	Parameter				*R*^2^
Igarashi model	Symbol	Best fit	2.5%	97.5%	0.66
	*a*	0.28	0.14	1.09	
	*b*	1.37	0.74	3.05	
	*d*	2117	1032	2989	

This study is the first to show the long-term diminishing trend of the ^90^Sr concentration through the physical decay of ^90^Sr and diffusion in the environment, as well as variations in the ^90^Sr concentration corresponding to changes in the discharge rate. The mathematical implementation based on assumptions from previous studies enables us to describe the time-dependency of the ^90^Sr-*Q* relationship. If the solute concentration gradient in the soil involves hydrochemical processes in the river, our model can be applied to other radionuclides besides ^90^Sr. Finally, this study is the first to examine the relationship between dissolved-phase radionuclides and stream discharge from a hydrochemical perspective, and highlights the applicability of this scheme to other radionuclides in rivers.

## Methods

Experiments were conducted at the Sakhan river in the CEZ (51.41°N, 30.00°E). This river lies within one of the sub-catchments of the Pripyat River, and has a total surface area of 186.9 km^2^ (Fig. 1). Annual rainfall in the CEZ is 500–600 mm/year. Monthly minimum and maximum temperatures are −6 °C and 19 °C, respectively. The catchment is largely covered by grassy meadow and wetlands. The forest area is occupied by coniferous and mixed forests^33^. The geological strata are composed of sandy quaternary deposits^33^. The topography is relatively flat. Local research institutes have been measuring the radionuclide concentrations in the local environment, including in soil and river water collected from various sites in the CEZ, over a 30-year period since soon after the CNPP accident. The ^90^Sr concentration and water discharge have been measured at the Sakhan river bridge monitoring station, located 7 km northwest of the nuclear power plant. The initial average ^90^Sr concentration in the soil was estimated to be 470.7 kBq/m^2^ (in 1986)^5^. The main long-term source of exchangeable and available ^90^Sr in the Sakhan catchment is non-oxidized FP^2–5^. The measurement data used in this study were obtained from two research institutes, the Ukrainian Hydrometeorological Institute (data period: 1990–1998) and the State Agency of Ukraine on Exclusion Zone Management and Chernobyl Ecocentre (data period: 1999–2016). Sampling was carried out in intervals of several weeks to several months in the initial phase. Continuous biweekly-interval sampling commenced in March, 1992. The ^90^Sr measurement error, available only for the period 1999–2016, was 10.9 ± 2.01% (mean ± S.D.). Water discharge data were obtained by integrating the cross-section observations of flow velocity and water depth from a concrete revetment under a bridge. To verify our estimates, flow rates were also measured directly using an electromagnetic current meter from March–May 2018, and the differences in the results were found to be insignificant.

## Supplementary information

Supplementary information.
