# Supplementary material for: Simulating dissolved 90Sr concentrations within a small catchment in the Chernobyl Exclusion Zone using a parametric hydrochemical model
Source: Sci Rep. 2020 Jun 17;10:9818. doi: 10.1038/s41598-020-66623-4 (PMC7299996; doi:10.1038/s41598-020-66623-4)
Supplement: Supplementary file 1 — Supplementary information. [file 41598_2020_66623_MOESM1_ESM.docx]

**Simulating dissolved ^90^Sr concentrations within a small catchment in the Chernobyl Exclusion Zone using parametric hydrochemical model**

**Yasunori Igarashi**^1,*^**, Yuichi Onda**^2^**, Jim Smith**^3^**, Sergey Obrizan**^4^**, Serhii Kirieiev**^5^**, Volodymyr Demianovych**^5^**, Gennady Laptev**^6^**, Dmitri Bugai**^7^**, Hlib Lisovyi**^6^**, Alexei Konoplev**^1^**, Mark Zheleznyak**^1^**, Yoshifumi Wakiyama**^1^**, and Kenji Nanba**^1^

^1^ Institute of Environmental Radioactivity, Fukushima University, Fukushima, Japan

^2^ Center for Research in Isotopes and Environmental Dynamics, University of Tsukuba

^3^ School of Earth & Environmental Sciences, University of Portsmouth, Portsmouth, UK

^4^ The Chornobyl Radiation and Ecological Biosphere Reserve, Chernobyl, Ukraine

^5^ Chernobyl Ecocentre, State Agency of Ukraine on Exclusion Zone Management, Chernobyl, Ukraine

^6^ Ukrainian Hydrometeorological Institute, Kiev, Ukraine

^7^ Institute of Geological Sciences, Kiev, Ukraine

^*^ Correspondence and requests for materials should be addressed to Y.I. (email: y-igarashi@ipc.fukushima-u.ac.jp)

1. **Description of Parametric Hydrochemical Model**

﻿

**Igarashi model**

This is a new conceptual model for simulating radionuclide concentrations in stream water, especially dissolved-phase ^90^Sr, whereby a *C*-*Q* relationship is derived by developing the idea of a distribution of the lateral flow of water across the vertical profile of soil water chemistry near the river^1^.

The key assumption in our model is that the distribution of the lateral flow of soil and the vertical profile of radionuclides in the soil provides a stream discharge and determines the radionuclide concentration in a stream. The radionuclide concentration profile in the soil is represented by the following modified equation:

$c\left( z,t \right)=C_{\mathrm{ref}}exp(\frac{dz}{t})$ (S1)

where *c*(*z*, *t*) is the concentration as a function of depth *z* and time *t* since fallout on the surface (26 April, 1986). *C*_ref_ is the long-term diminishing trend of the ^90^Sr concentration. *a* and d*d* are model coefficients. The *z*-axis has negative values below the ground surface, and intersects the ground surface at *z*_0_.

As in a previous study, the flow can be described as a function of depth as follows:

$q\left( z \right)=a\exp\left( bz \right)$ (S2)

where *a* and *b* are parameters. If we can assume that the solution concentration in the stream water is formed by a lateral flow passing through the vertical concentration profile of ^90^Sr in the soil, the dissolved ^90^Sr concentration, which represents the values at the catchment outlet, can be calculated from the load and the discharge rate as follows:^4^

$C=\frac{L}{Q}=\frac{\int_{z0}^{z1} q\left( z \right)c\left( z,t \right)dz}{\int_{z0}^{z1} q\left( z \right)dz}$ (S3)

﻿Here, the total mass flow rate of this constituent, i.e. the constituent load *L* [MT^-1^], in the discharge is then the integral of these lateral mass fluxes over depth from a certain base level (bedrock), *z*_0_, to the groundwater table, *z*_1_ (S3). Lateral fluxes above the groundwater table and below *z*_0_ are neglected. Under the assumption that all water passes the catchment soil before reaching the stream, the water-flux integral equals the discharge *Q* [L^3^ T^-1^]. The concentration in the stream, *C* [ML^-3^], can then be computed as the mass flux integral divided by the water-flux integral (S3).

Thus, ﻿dissolved ^90^Sr concentration loads can be calculated as follows:

$L=\int_{z_{0}}^{z_{1}} a\exp\left( bz \right)\times C_{\mathrm{ref}}exp(\frac{dz}{t})dz$ (S4)

Rewriting equation (S2) results in a power law in which the dissolved ^90^Sr concentration is directly related to discharge *Q*:

$z=b^{-1}\ln\left( {bQ}/a \right)$ (S4)

$dz=\left( bQ \right)^{-1}dQ$ (S5)

$$L=aC_{\mathrm{ref}}\int_{z_{0}}^{z_{1}} \exp\left( bz \right)exp(\frac{dz}{t})dz$$

$$L=C_{\mathrm{ref}}\int_{0}^{Q_{1}} exp(\frac{d\ln\left( {bQ}/a \right)}{bt})dQ$$

$$L=C_{\mathrm{ref}}\int_{0}^{Q_{1}} \left( \frac{bQ}{a} \right)^{d/{bt}}dQ$$

$$L=C_{\mathrm{ref}}\left[ \frac{btQ\left( \frac{bQ}{a} \right)^{d/{bt}}}{bt+d} \right]_{0}^{Q_{1}}$$

$$L=\frac{C_{\mathrm{ref}}}{bt+d}btQ\left( \frac{bQ}{a} \right)^{d/{bt}}$$

Thus, the ^90^Sr concentration in the stream, which is defined by an integral expression, can be solved analytically to give:

$C\left( Q,t \right)=\frac{C_{\mathrm{ref}}bt}{bt+d}\left( \frac{bQ}{a} \right)^{d/{bt}}$ (S6)

Here, all parameters have positive values. ﻿It is interesting to note that this type of power-law function also appears as an empirical means of describing the log(*C*)-log(*Q*) relationship. In mathematical terms, it has been clarified that the relationship between ^90^Sr and *Q* in a stream has a log-log relationship. Another interesting point is that the ^90^Sr concentration profile in soil already includes the effect of radioactive decay; thus, our Igarashi model is independent of *C*_ref_. The model parameters were estimated through a Markov chain Monte Carlo calibration procedure using DREAMZS^2,3^. The "*credible interval*" for each model parameter is described in Table 2; the probability density function for each parameter is also given. The search range of the parameters in equation (S6) are presented in the following table.

Table. List of calibration parameters

| ﻿Symbol | Lower bound | Upper bound |
| --- | --- | --- |
| *a* | 0 | 5 |
| *b* | 0 | 5 |
| *d* | 0 | 4000 |

1. **Estimated model parameters**

The model parameters were estimated through a Markov chain Monte Carlo calibration procedure. The posterior distributions of the parametric model parameters are shown in Figure S1.

**
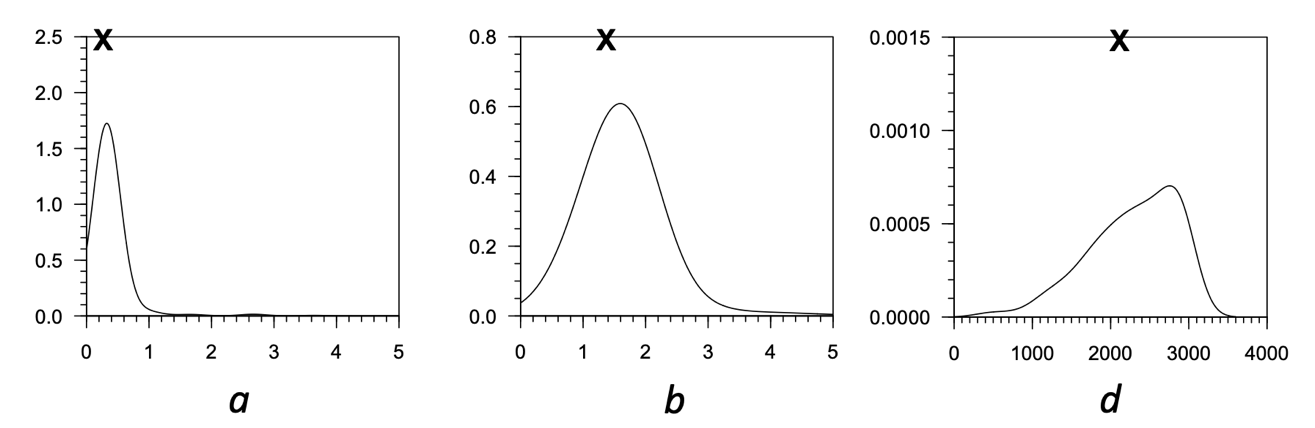
**

**Supplemental Figure S1** Posterior distributions of parametric model parameters for Igarashi model. **X** indicates the maximum a posteriori value (= best fitted parameter). The units on the y axes are probability density functions of the x axes. The maximum a posteriori method is a Bayesian-based approach for estimating a distribution and model parameters that best explain an observed dataset.

**References**

1. ﻿Seibert, J. *et al.* Linking soil- and stream-water chemistry based on a Riparian Flow-Concentration Integration Model. *Hydrol. Earth Syst. Sci.* **13**, 2287-2297 (2009).
2. ter Braak, C.J.F. and Vrugt, J.A. Differential evolution Markov Chain with snooker updater and fewer chains, *Stat. Comput.* **18**(4), 435–446 (2008)
3. Vrugt, J.A. *et al*., Accelerating Markov Chain Monte Carlo Simulation by Differential Evolution with Self-Adaptive Randomized Subspace Sampling. *Int. J. Nonlinear Sci. Numer. Simul.* **10**, 273-290 (2009).
